# Workers with Suspected Diagnosis of Silicosis: A Case Study of Sarcoidosis Versus Siderosis

**DOI:** 10.3390/healthcare11121782

**Published:** 2023-06-16

**Authors:** Diemen Delgado-García, Patricio Miranda-Astorga, Ashley Delgado-Cano, Juan Gómez-Salgado, Carlos Ruiz-Frutos

**Affiliations:** 1 Department of Research and Postgraduate, Universidad de Aconcagua, Los Andes 2102660, Chile; 2 School of Medicine, Neurology and Psychiatry, Universidad de Texas Rio Grande Valley, Edinburg, TX 78539, USA; 3 Departament of Occupational Health, Instituto de Salud Pública de Chile, Santiago 7780050, Chile; 4 School of Medicine, Universidad Andrés Bello-Viña del Mar, Valparaíso 2520000, Chile; 5 Department of Sociology, Social Work and Public Health, Faculty of Labour Sciences, University of Huelva, 21007 Huelva, Spain; 6 Safety and Health Postgraduate Programme, Universidad Espíritu Santo, Guayaquil 092301, Ecuador

**Keywords:** diagnosis, silicosis, sarcoidosis, siderosis

## Abstract

Silicosis is one of the most important occupational respiratory diseases worldwide, hence the importance of making a correct diagnosis. Diagnosis is commonly based on radiological findings according to the ILO International Classification of Radiographs of Pneumoconioses and occupational exposure. High-resolution computed tomography is indicated for differential diagnosis. This article presents two cases with an initial diagnosis of silicosis that ended up being diagnosed as sarcoidosis and siderosis, respectively. The first case was a 42-year-old male who worked as a crushing operator in an underground copper and molybdenum mine for 22 years. He had a history of exposure to silicon dioxide and was asymptomatic. X-rays did not distinguish silicosis or siderosis, but histological findings (open lung biopsy) allowed for a diagnosis of sarcoidosis. The second case was a 50-year-old male who had worked as a welder in a molybdenum filter plant, an open pit mine since 2013; he spent the previous 20 years as a welder in an underground copper mine, with exposure to silicon dioxide and was symptomatic. The first radiograph showed opacities that were compatible with pulmonary silicosis. A subsequent high-resolution computed tomography and lung biopsy showed a pattern of pulmonary siderosis. Due to the similarities in the radiographs of these three diseases, greater emphasis must be placed on the differential diagnosis, for which a complete occupational and clinical history is important in order to provide clues for the performance of complementary tests to avoid misdiagnosing.

## 1. Introduction

Silicosis is caused by the inhalation of silicon dioxide (SiO_2_). These particles cause inflammation and fibrosis in the lungs, leading to progressive, irreversible, and potentially disabling lung disease. It is one of the most important occupational respiratory diseases worldwide [1].

The exact pathogenic mechanism of silica particles is still one of the major problems in toxicology. Recent developments in silica surface chemistry, cell biology, and toxicology have provided new insights into the variable reactivity of silica particles owing to their surface configuration and chemical characteristics [2,3].

Based on this pathophysiological mechanism, four clinical forms are currently recognised: (A) simple chronic silicosis resulting from long-term exposure (more than 20 years) to low amounts of silica dust, sometimes even after exposure has ceased [4]. (B) Acute silicosis is a rapidly progressive clinical condition after intense exposure to free silica [5]. (C) Acute silicoproteinosis, which is a rare variant manifested as a bilateral perihilar alveolar infiltrate with a ‘ground-glass’ appearance [6]. Additionally, (D), there is accelerated silicosis, which is another under-defined clinical form that occurs after exposure to higher amounts of silica over a shorter period (5–15 years) [7].

The diagnosis of silicosis is made on the basis of exposure and radiological findings, according to the International Labour Organisation (ILO) Encyclopedia [8] and Classification of Radiographs of Pneumoconioses [9]. The ILO system classified parenchymal opacities by size, shape, and profusion through a comparison of 22 chest radiographs, which were selected after international trials in order to illustrate the rules of the intermediate profusion categories of small, rounded opacities and to provide examples of the rules for categories A, B, and C for large opacities (silicomas) [9,10]. In simple chronic silicosis, ILO chest radiographs show a nodular pattern of small round opacities [11], which are symmetrically distributed and predominantly in the upper zone. This complicated form of silicosis is characterised by the presence of masses originating from the confluence of the so-called nodules, also known as progressive massive fibrosis [12].

On other occasions, there is a diffuse interstitial pattern without the typical round opacities [13]. As time progresses, they increase in size and become confluent; the small round opacities may disappear, and the hilar structures can also be modified, leaving hyper-translucent lung areas in the periphery and lower lung zones [14]. Hilar and mediastinal lymph nodes are often enlarged and calcified, sometimes with a characteristic eggshell pattern [15], similar to the presence of sarcoidosis. The interpretation of ILO chest radiography improves as the degree of silicosis increases, but a significant proportion of patients with moderate or severe silicosis classified by histology may not be diagnosed with this radiological technique [16]. High-resolution non-contrast chest computed tomography (HRCT), which is more sensitive than a chest X-ray with the ILO technique, often shows nodular changes in the lung parenchyma and very frequently pleural, mediastinal, and hilar alterations in simple silicosis [17]. Spirometry may be normal in simple silicosis; in advanced stages, there may be degrees of restrictive or obstructive patterns, which depend largely on the associated comorbidity [18]. A lung biopsy is not recommended to confirm the diagnosis of silicosis but could be performed to exclude other pathologies in the case of clear doubt [19]. Bronchoscopy and bronchoalveolar lavage could be useful in the diagnosis of silicoproteinosis [20] and silico-tuberculosis [21].

Indeed, an exhaustive clinical differential diagnosis of sarcoidosis [22] and siderosis [23] is necessary since the radiological patterns of these diseases can be similar. Sarcoidosis is a multisystem granulomatous disease of unknown aetiology whose immunological mechanism has not yet been identified [24]. This fact has led some authors to establish the diagnosis of sarcoidosis from the exclusion of other sarcoid-like diseases, according to its clinicopathological findings [25]. Siderosis is a benign pneumoconiosis that develops as a result of exposure to metallic iron or iron oxide dust [26]. Occupations that can lead to the development of pulmonary siderosis include mining, welding, steel manufacturing, iron oxide manufacturing, grinding wheel manufacturing, and silver jewellery manufacturing [27].

This article presents two cases of patients diagnosed with silicosis, according to the ILO International Classification of Radiographs of Pneumoconioses [28], and who were eventually diagnosed with other pathologies. After the radiographic examination, lung biopsy results provided the gold standard in both cases.

## 2. Materials and Methods

In this study, a differential diagnosis of silicosis, sarcoidosis, and siderosis was approached through the clinical follow-up of two patients. A study of their occupational history was carried out, which was completed with a radiological study and HRCT. Subsequently, a functional respiratory study was performed and contrasted with histological findings in an anatomopathological study.

This study was approved by the Research Committee of the Universidad de Aconcagua. All study participants signed an informed consent form. We confirm that all methods were performed in accordance with the relevant guidelines and regulations.

## 3. Results

### 3.1. Presentation of Cases

#### 3.1.1. Case 1

**Figure 1:**
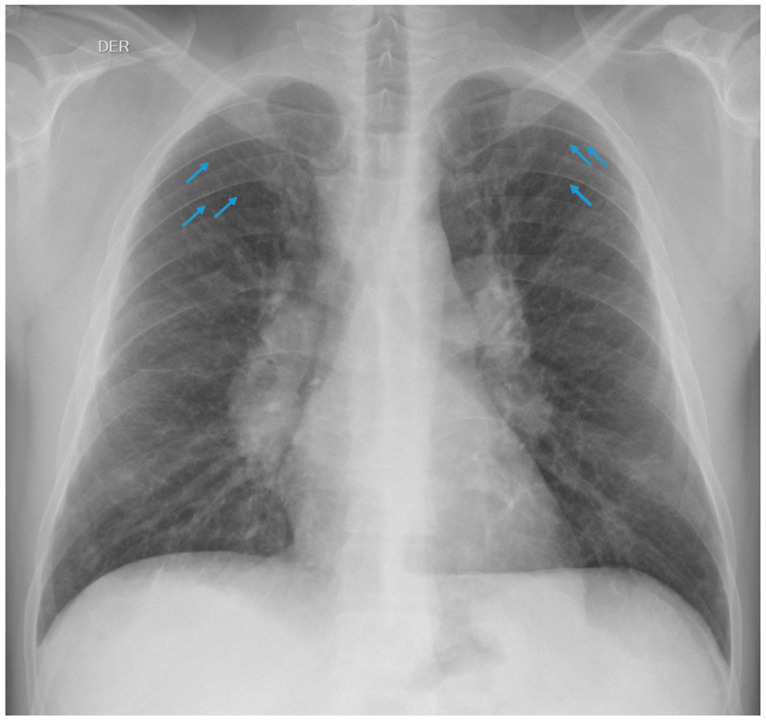
Small round opacities of type p/q, 1/0 profusion.

A 42-year-old man who had been working as a crusher operator in an underground copper and molybdenum mine for 22 years was examined. His occupational history confirmed exposure to silicon dioxide (SiO_2_), he was asymptomatic, and a chest X-ray was taken according to the International Classification standard of the International Labour Organisation, in accordance with the medical surveillance programme defined by his employer and the occupational health insurance. He was informed by the reading panel responsible for this technique, which was composed of two certified occupational physicians, of the presence on the X-ray of small round opacities of type p/q, 1/0 profusion, in the upper 1/3 of both hemithoraxes, which were compatible with pulmonary silicosis (Figure 1).

**Figure 2:**
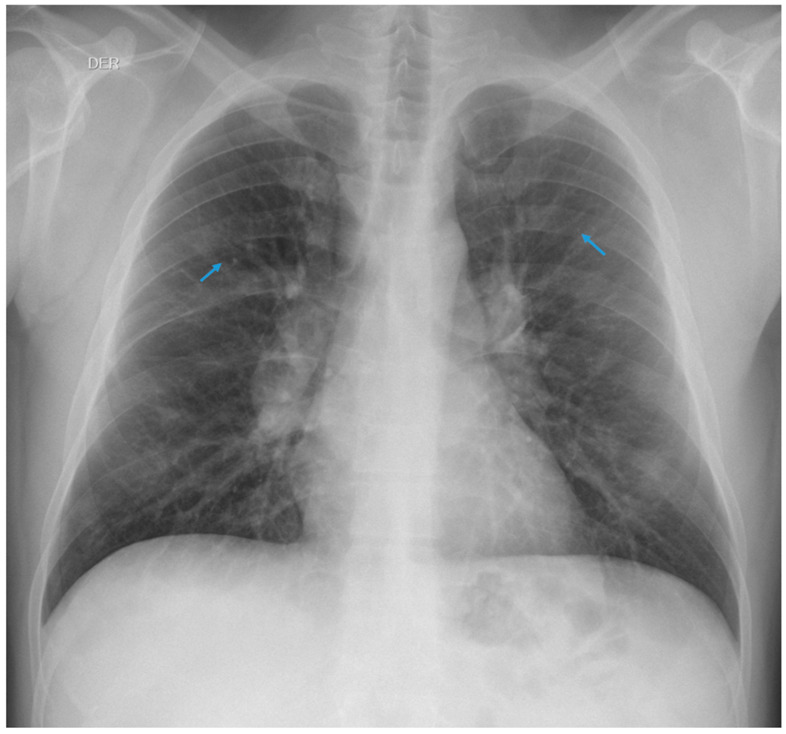
Small round opacities of type p/q, 0/1 profusion.

As part of his previous history, 2 years earlier, the patient brought in a reading record taken with this technique reporting the presence of small round opacities of type p/q, 0/1 profusion, in the upper 1/3 of both hemithoraxes. These minimal radiographic changes were considered inconsistent for suspicion of pulmonary silicosis in this previous evaluation (Figure 2).

**Figure 3:**
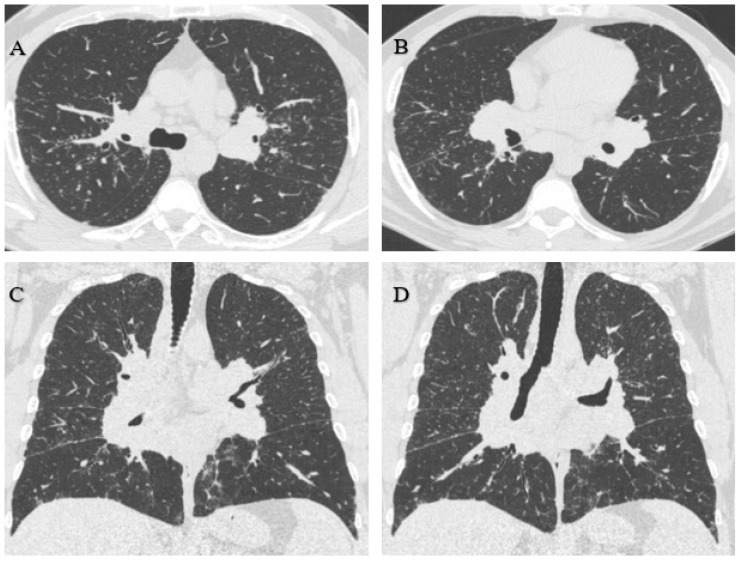
(**A**,**B**) Innumerable diffusely distributed perilymphatic nodules. (**C**,**D**) Confluent mediastinal and perihilar adenopathies of up to 3.2 cm.

Considering the actual and the older radiographs, an HRCT was indicated, which showed innumerable perilymphatic nodules of diffuse distribution that could be associated with confluent mediastinal and perihilar adenopathies of up to 3.2 cm; this is characteristic of sarcoidosis. In the posterior basal segment of the right lower lobe, a confluent ring-shaped opacity of 3.1 cm was observed (Figure 3).

**Figure 4:**
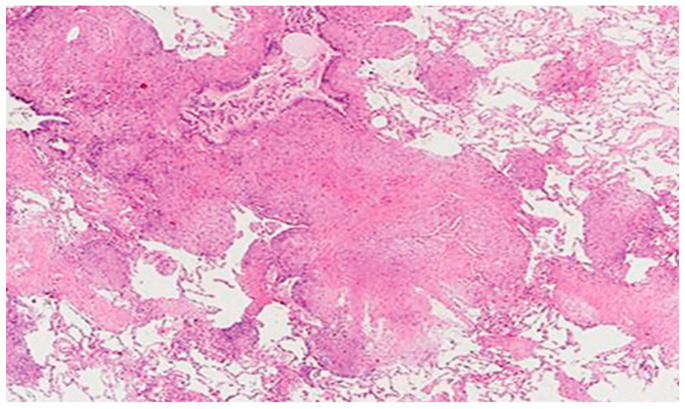
Nodular areas of non-necrotising granulomatous histiocytic proliferation.

The radiological findings did not allow a diagnostic distinction to be made between silicosis and sarcoidosis. Therefore, an anatomopathological study (open lung biopsy) was requested, showing lung parenchyma with four nodular areas of non-necrotising granulomatous histiocytic proliferation, including moderate lymphocytes and some multinucleated giant cells. No swirling fibrosis was observed, nor birefringent spicules in the polarised light. These histological findings favoured the diagnosis of sarcoidosis (Figure 4).

A functional respiratory study was not performed, nor was a mycobacterial study [29] through the PCR technique or Grocott stain for fungi (the gold standard to rule out infection), as the patient did not present clinical symptoms or impairment of the general condition.

#### 3.1.2. Case 2

**Figure 5:**
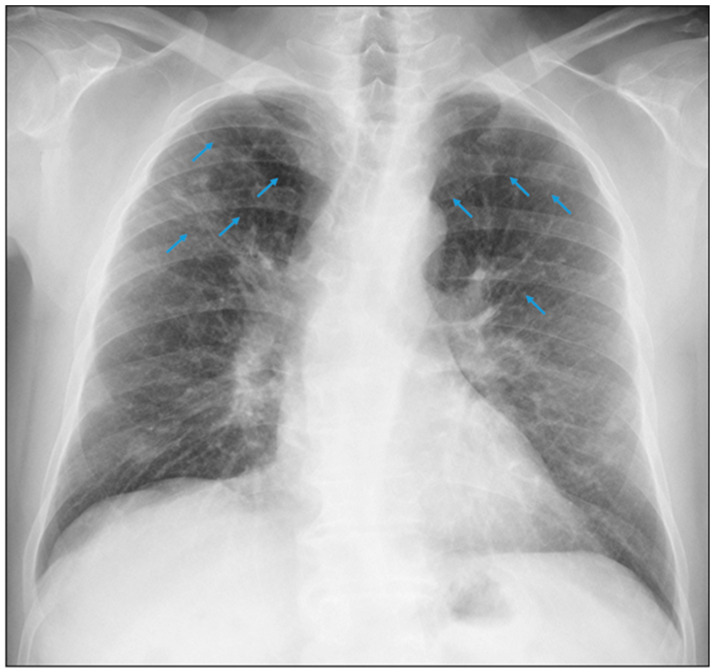
Small round opacities of type p/q, 1/2 profusion.

A 50-year-old male who had been working as a welder in a molybdenum filter plant of an opencast mining company since 2013 was examined. His occupational history confirmed previous exposure to silicon dioxide (SiO_2_) for 20 years while performing the same job as a welder but in an underground copper mine. According to his occupational history, he had not been exposed to silicon dioxide (SiO_2_) in his last job. He had been presenting with a dry cough for 4 months, with chest pain and dyspnoea on exertion for the last 2 months. For this reason, he underwent a chest X-ray according to the International Classification of the ILO since, according to the worker, exposure to dust and fumes at work was the cause of his symptoms. The radiograph was reported by the reading panel responsible for this technique, which was composed of two certified occupational physicians. The radiograph showed small round opacities of p/q type, 1/2 profusion, in the upper 1/3 of both hemithoraxes, which is compatible with pulmonary silicosis (Figure 5).

**Figure 6:**
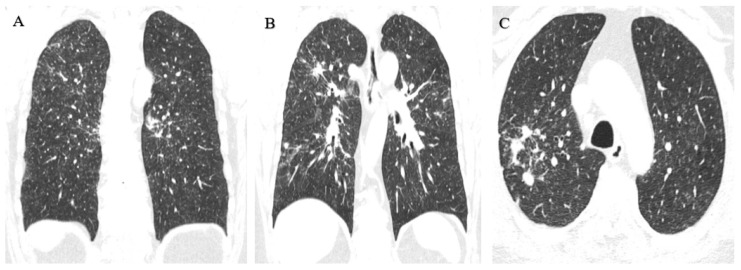
(**A**) Diffuse, segmentally distributed micronodular disease more predominant in the middle thirds. (**B**,**C**) Larger sized qualified nodules in the right upper lobe, other dense and irregular nodules in both lower lobes, with the most significant in the paraspinal cortex of the apical segment of the right lower lobe of 9 mm.

There was no previous record of an X-ray with this technique, but there was an HRCT taken 3 months earlier by his private health service for presenting with a dry cough. It showed diffuse micronodular disease in segmental distribution, more predominant in the middle thirds, with some more dense and irregular images and thickening of the bronchovascular axes, as well as an irregularity of the subpleural connective tissue; larger qualified nodules were located in the right upper lobe and other dense and irregular nodules in both lower lobes. The most significant one was in the paraspinal cortex of the apical segment of the right lower lobe, measuring 9 mm, with hilar thickening by discreetly enlarged ganglia. In the mediastinum, there were a few nodes smaller than 1 cm. These findings did not allow a diagnostic distinction to be made between pneumoconiosis and the possibility of an associated neoplasm (Figure 6).

**Figure 7:**
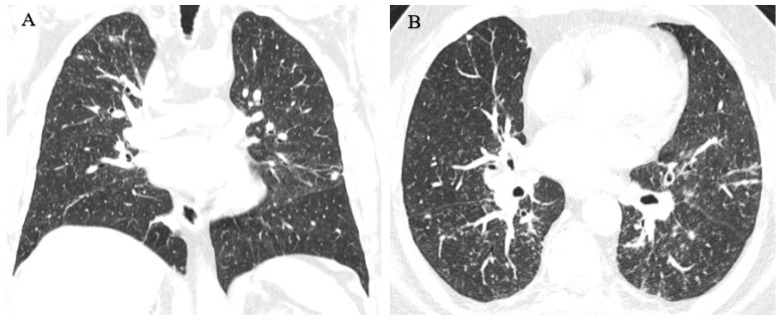
(**A**) Innumerable ground-glass nodules of perilymphatic and centrilobular distribution. (**B**) Some larger nodules in the peribronchovascular situation and with linear/reticular opacities.

For this reason, a new HRCT scan was requested 2 months later, which showed innumerable ground-glass opacity nodules in the lung parenchyma with a perilymphatic and centrilobular distribution, including some larger nodules in a peribronchovascular situation and with linear/reticular opacities. This pattern is compatible with pulmonary siderosis (Figure 7).

**Figure 8:**
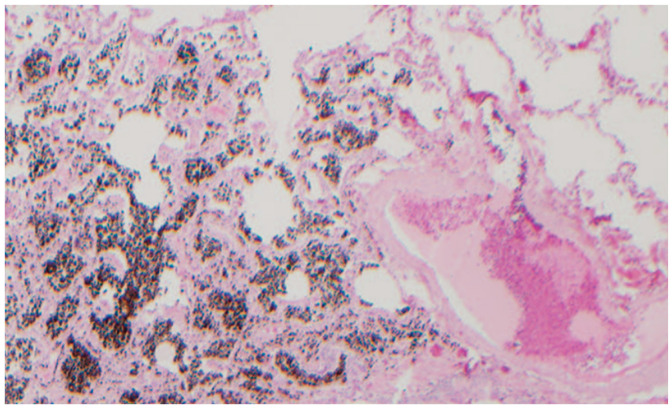
Iron content in macrophages and peribronchovascular interstitium.

An anatomopathological study was performed (open lung biopsy), which confirmed the presence of high iron content in the macrophages and in the peribronchovascular interstitium (Figure 8). A functional respiratory study was completed, concluding with a mild restrictive spirometric pattern.

## 4. Discussion

In the early stages of silicosis, there may be no obvious indicators, and the disease may be asymptomatic. Silicosis develops slowly over a long period of exposure to crystalline silica. In more advanced stages of the disease, a persistent cough, shortness of breath, and fatigue usually occur, and these symptoms may become more evident [5,6].

In some cases, siderosis can also be asymptomatic, and radiographs may show the presence of small dotted or line-shaped opacities in the lung fields [23].

Sarcoidosis can be asymptomatic in its early stages. In many cases, sarcoidosis is diagnosed incidentally during routine medical exams or imaging studies, even when the patient has no obvious symptoms [22,30].

In the two cases presented, the differential diagnosis of silicosis versus sarcoidosis [30] and siderosis [31] is of clinical interest. Radiological features, along with the patient’s occupational history, demonstrated exposure to silicon dioxide (SiO_2_), and this led to a diagnosis of silicosis according to the ILO International Classification of Radiographs of Pneumoconioses [11]. In the first case, the worker had no respiratory or other organ-related symptoms. The appearance of non-caseating granulomatous inflammation [32] in the lung biopsy, together with the imaging characteristics provided by HRCT, made the initial diagnosis of silicosis unlikely and pointed toward a diagnosis of sarcoidosis. However, the link between silicosis and sarcoidosis is interesting, as retrospective studies have shown an increased risk of sarcoidosis in workers with occupational exposure to silicon dioxide (SiO_2_) [33].

In the second case, the worker presented with respiratory symptoms [34], which is very rare in the early stages of silicosis [35], as well as the appearance of iron-laden macrophages in the lung biopsy [36] when together with the characteristics of the images provided by the HRCT, and his work history as a welder. This made the initial diagnosis of silicosis unlikely and pointed toward a diagnosis of siderosis.

It is known from previous studies that silicosis can have atypical presentations [37,38,39]. The identification of small round opacities in terms of their type and profusion, according to the methodology of the ILO [11], is extremely similar for silicosis, sarcoidosis, and siderosis. Hence, the importance of a differential diagnosis is evident [40].

A differential diagnosis with other pathologies is highly recommended, including pulmonary tuberculosis, pulmonary aspergillosis, pulmonary metastasis, hypersensitivity pneumonitis, coal workers’ pneumoconiosis, kaolin pneumoconiosis, and berylliosis. Silicosis and tuberculosis are the two diseases that remain high on the list of occupational health priorities in low-income countries and still occur in some high-income countries [41]. Tuberculosis and aspergillosis were ruled out due to the characteristics of radiological images; however, a molecular biological study was not carried out.

Pulmonary involvement is characterised by radiographic images of nodular opacities from 2 to 3 mm and is often distributed diffusely and uniformly in both lungs, with a slight predominance in the lower lobes.

Chronic necrotising pulmonary aspergillosis is a rare disease that tends to affect people with underlying lung disease or who are mildly immunocompromised. It is an exceptional complication of silicosis, and its diagnosis requires a high degree of suspicion as it resembles other diseases, such as tuberculosis and neoplasia, which are more frequent complications in these patients [42]. The presence of irregular borders may suggest a primary lung tumour rather than metastasis.

Hypersensitivity pneumonitis diagnosis is basically determined by the identification of allergen (exposure and antibodies), dyspnoea and crackles, a functional restrictive pattern, compatible tomographic image, almost always the presence of ground glass and bronchocentric nodules, lymphocytosis in the bronchioloalveolar lavage, and the histological pattern of lymphocytic interstitial inflammation and ill-defined granulomas [43]. Coal workers’ pneumoconiosis is caused by cumulative exposure to silica dust in coal mines and is radiologically characterised by nodular opacities, which are either associated or not with centrolobular emphysema [44]. Kaolin pneumoconiosis is a mixed-dust pneumoconiosis, which is caused by the inhalation of silica and kaolin (hydrated aluminium silicate) and is characterised by early onset and rapid progression. Radiologically, nodular opacities can be observed, which are initially present in the lung apices [45].

Berylliosis is a chronic and occupational lung disease that is caused by a delayed hypersensitivity reaction to beryllium [46]. The acute form resembles chemical pneumonia, and the chronic form resembles sarcoidosis; hence, complementary immunological tests may be needed. Finally, pneumoconiosis due to the inhalation of other metals, such as tin, antimony, and barium, can give radiological images similar to siderosis. These metal-mediated pneumoconioses in which there is no fibrotic pathological reaction are often called [47] ‘benign pneumoconioses’.

This study had a number of limitations. The first refers to the description of clinical cases since this study focused on differential diagnosis and image analysis. No invasive treatments or techniques were used to counteract the disease. Secondly, the sample was reduced to two workers working in unfavourable conditions and with dangerous products. Therefore, the conclusions obtained from this study for occupational health should be considered from the comparison of working conditions and the analysis of preventive measures in the labour sector. Finally, readers are reminded that the objective of this study was to expose two cases that were misdiagnosed, as well as to expose the differential factors that confirmed both diagnoses.

## 5. Conclusions

A complete occupational and clinical history is essential to avoid misdiagnosis in cases of suspected silicosis, given its implications for the therapeutic approach, evolution, and prognosis of the workers involved. The similarity in radiographic readings requires greater emphasis on the differential diagnosis between these pathologies. A high-resolution non-contrast computed tomography of the chest should be performed, and a lung biopsy should be requested in cases of comorbidity, whether such diagnostic tests are available. In addition, differential diagnosis with other pathologies is suggested, and siderosis does not exclude concomitant silicosis.

## Data Availability

All data generated during this study are available within this article.
